# Chemometric Study of Trace Elements in Hard Coals of the Upper Silesian Coal Basin, Poland

**DOI:** 10.1155/2014/234204

**Published:** 2014-05-22

**Authors:** Adam Smoliński, Przemysław Rompalski, Krzysztof Cybulski, Jarosław Chećko, Natalia Howaniec

**Affiliations:** ^1^Department of Energy Saving and Air Protection, Central Mining Institute, Plac Gwarków 1, 40-166 Katowice, Poland; ^2^Department of Solid Fuels Quality Assessment, Central Mining Institute, Plac Gwarków 1, 40-166 Katowice, Poland; ^3^Central Mining Institute, Experimental Mine “Barbara”, Podleska Street 72, 43-190 Mikołów, Poland; ^4^Department of Geology and Geophysics, Central Mining Institute, Plac Gwarków 1, 40-166 Katowice, Poland

## Abstract

The objective of the study was the analysis of trace elements contents in coals of the Upper Silesian Coal Basin (USCB), which may pose a potential threat to the environment when emitted from coal processing systems. Productive carbon overburden in central and southern zones of the USCB is composed mostly of insulating tertiary formations of a thickness from a few m to 1,100 m, and is represented by Miocene and Pliocene formations. In the data study the geological conditions of the coal seams of particular zones of the USCB were taken into account and the hierarchical clustering analysis was applied, which enabled the exploration of the dissimilarities between coal samples of various zones of the USCB in terms of basic physical and chemical parameters and trace elements contents. Coals of the northern and eastern zones of the USCB are characterized by high average Hg and low average Ba, Cr, and Ni contents, whereas coals of southern and western zones are unique due to high average concentrations of Ba, Co, Cu, Ni, and V. Coals of the central part of the USCB are characterized by the highest average concentration of Mn and the lowest average concentrations of As, Cd, Pb, V, and Zn.

## 1. Introduction

Coal is considered to be the dominant fossil fuel in terms of the world energy supplies security. Over 90% of Polish and 40% of the world power generation is based on coal [1]. Poland is the eighth world coal producer (71.34 mln Mg/year of hard coal and 64.30 mln Mg/year of lignite in 2012) [2]. The processes of thermochemical conversion of coal are, however, the sources of contaminants, such as carbon dioxide, nitrogen oxides, sulfur oxides, dust, and trace elements [3–5]. Clean Coal Technologies (CCT) are developed to mitigate the negative environmental impact of coal processing in the energy sector. Emissions of hazardous elements from coal combustion process have been extensively studied in recent years [6–13]. Recognition of the modes of occurrence of trace elements in coal is crucial in understanding their chemical, thermal, and environmental behaviors in the processes of coal treatment and thermochemical conversion [14, 15]. The information on geochemical characteristics of coal seam as well as the chemical and elemental composition is fundamental for efficient utilization of coal and reduction of its negative environmental impact [16]. The behavior of trace elements depends on coal types [17, 18] and on the operating parameters of the combustion process. High combustion temperatures and reducing atmosphere promote the release of trace metals [19].

The main objective of the study was to investigate the differences between coals provided by coal mines located in various zones of the Upper Silesian Coal Basin (USCB), the largest hard coal basin in Poland. The relationships between the basic physical and chemical parameters and trace elements concentrations in hard coal samples taking into account the geological conditions of the USCB were also investigated. For that purpose the hierarchical clustering analysis (HCA) [20–26] was applied complemented with a color map of the studied data set, which allowed more in-depth interpretation of the clustering tendency between hard coal samples of various zones of the Upper Silesian Coal Basin.

## 2. Geological Settings

The Upper Silesian Coal Basin is located on the area of Silesian voivodeship and the eastern part of Malopolska voivodeship. Productive carbon overburden in central and southern zones of the USCB is composed mostly of insulating tertiary formations of thickness from a few m to 1,100 m [27]. It is represented by Miocene and, on a small area, Pliocene formations. Mining has been started here in the mid-17th century. Nowadays, there are 120 explored and documented hard coal deposits in the USCB, including 46 deposits in operation in 32 coal mines, 34 abandoned deposits, and 40 undeveloped deposits.

**Figure 1:**
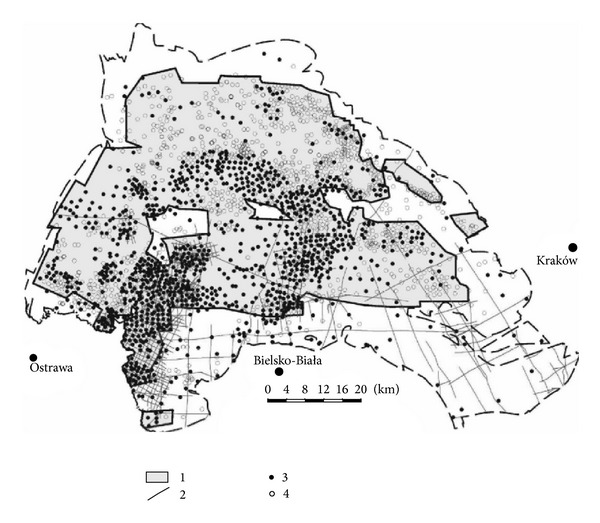
Geological exploration of the Upper Silesian Coal basin 1—documented area, 2—seismic profiles, 3—wells of the depth of at least 1,000 m, and 4—wells of a depth of 500–1,000 m [28].

The total documented area of the USCB is 3295 km^2^. The operated coal deposits cover 1,030 km^2^ (approximately 18% of the USCB area) and depleted or abandoned deposits: 706 km^2^ (approximately 12%). Most of the deposits in operation or abandoned are located in the northern and western parts of the basin. The remaining, undocumented areas of the basin cover southern, eastern, and to a less extent, the northern parts [28]. This area is less geologically explored than the deposits in operation (Figure 1).

The USCB is surrounded by a range of Upper Carboniferous coal-bearing formations and partially by fault lines. To the west it is limited by Lower Carboniferous folded flysch formations.

The north-eastern border is buried under Permian and Triassic formations. Below the Carboniferous formations there are Lower Paleozoic formations, covered with Devonian and Lower Carbon limestone formations (Carboniferous limestone). The south, erosion border, goes under the Carpathian flysch thrust. Here, the metamorphic Precambrian formations may be found, and over them, the Cambrian and Devonian formations. The Carboniferous roof is located here at the depth of 2,000–3,000 m.

The base of the USCB constitutes Precambrian, Cambrian, Devonian, and, partially, younger Carboniferous rocks [27].

The structure of productive Carboniferous formations in the basin is asymmetric. The Carboniferous series is characterized by a dual structure. The lower part, Paralic series, is placed on marine sediments. The upper part, continental deposits of the Upper Silesian sandstone series, mudstone series, and Cracow sandstone series are placed on paralic hiatal surface.

Paralic series are classified to Namur. It is composed of clastic sediments composed mainly of fine- and medium-grained sandstones (20–50% of the series) and biogenic sediments (3-4%). The thickness of paralic series in the eastern part of the USCB is 200 m, and in the western part almost 3,800 m. 110 coal deposits of a thickness of 1–1.5 m were distinguished in the paralic series profile.

The USCB sandstone series is the first series of so called continental deposits of productive Carboniferous rocks. Here, mainly sandstones, conglomerates, thick coal layers and also fine-grained sandstones and mudstones can be found. Coarse clastic deposits constitute up to 70–90% of the profile. There are approximately 60 coal deposits in the Upper Silesian sandstone series of the thickness of 4–8 m [27]. The highest thickness value is reported for coal deposit no. 510, named Reden.

The maximum thickness of the mudstone series in the western part of the basin is up to 2,000 m and it is reduced to 150 m in the eastern part. This is the dominant series among the continental productive Carboniferous formations in terms of the area of fine-grain formations: mudstone, mudstone with sandstone, and rarely claystone. Sandstones, mostly fine-grained, constitute only 15–25% of the profile thickness. There are also numerous thin and variable coal layers of the thickness rarely exceeding 1.5 m. The total of 160 coal layers was determined in the series, of which 70 are of economic importance. The total share of coal in the series profile is 5–7%. It is classified to Westphalian A and lower Westphalian B strata.

Cracow sandstone series is the youngest formation of productive Carboniferous. It is characterized by homogenous structure and is composed mostly of coarse-grained sediments (approximately 70% of the profile, locally up to 90%) of coarse- and medium-grained sandstone of large thickness, separated with intervals of fine-grained sediments, among which coal seams are located. The maximum thickness of the series is 1,140 m in the Libiąż area. The area of this series is the easternmost and is classified to Westphalian B–D. Coal layers are sparse but of considerable thickness, even of 6-7 m. In the profile 40 coal layers were distinguished, of which 20 were of economic importance.

**Figure 2:**
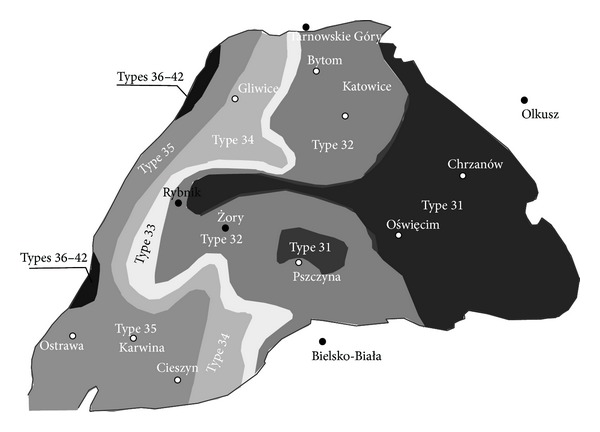
Distribution of coal types on USCB Carboniferous roof [33].

There are horizontal and vertical variations in coal types in the USCB [29], depending mainly on a coal seam depth and location within the USCB area. Steam coals of types 31–33 (according to the Polish Standard PN-82/G-97002) can be found on a considerable area of the basin (Figure 2). In the main basin and on the area between Oświęcim and Rybnik these types of coal are deposited at a depth below 1,000 m. The average ash content is 13% and in coal seams of particular series it varies between 11 and 16%. The average total sulfur content is between 0.59 and 2.3%. Cocking coals of types 34-35 are located in the south-western part of the USCB (Figure 2). Coal types 36–42 are rare and are situated in the western part of the USCB.

The documented balance resources of hard coal in Poland are estimated to be on the level of 48,225 million Mg (see Table 1). Polish hard coal deposits belong to the Carboniferous Euro-American province, which is represented in Europe by the belts of paralic and limnic basins [2]. Coal resources are located in three basins: Upper Silesian Coal Basin (USCB), Lublin Coal Basin (LCB), and Lower Silesian Coal Basin (LSCB). The USCB is the largest coal basin in Poland with the area of 5,600 km^2^ and over 80.2% of total domestic hard coal deposits. It may be divided into five zones: the northern, southern, eastern, western, and central. The LCB covers an area of 9,100 km^2^ with 20.9% of the total domestic hard coal deposits. The resources of the LSCB, estimated to be of approximately 359 million Mg, are located on the area of 350 km^2^ and have been abandoned for technical and economic reasons.

## 3. Materials and Methods

**Figure 3:**
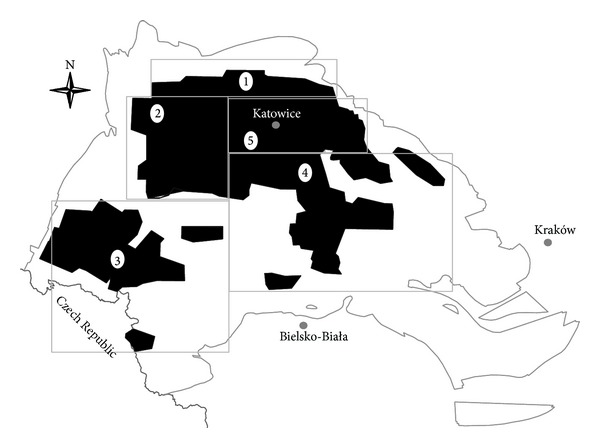
Division of the explored area: 1—north zone, 2—west zone, 3—south zone, 4—east zone, and 5—central zone.

The explored area was divided into five zones (Figure 3), including the following lithostratigraphic members:

- northern zone, including paralic series, Upper Silesian sandstone series, and mudstone series with abandoned coal mines,
- western zone, including paralic series with upper marginal beds, Upper Silesian sandstone series with saddle series, and mudstone series with rudzkie and orzeskie coal layers,
- southern zone includes marginal beds (paralic series), Upper Silesian sandstone series, and mudstone series with rudzkie and orzeskie coal layers,
- eastern zone includes Cracow sandstone series with łaziskie and libiąskie coal layers,
- central zone covers lower part of Cracow sandstone series with łaziskie coal layers, mudstone series, Upper Silesian sandstone series, and paralic series.

Hard coals samples were provided by selected coal mines located in the USCB. 9 samples were taken from coal mines located in the northern zone, 6 samples from the southern zone, 9 samples from the eastern zone, 7 samples from the western zone, and 13 samples from the central zone of the USCB, respectively. The samples were collected in accordance with the standard PN-90/G-04502 from various coal seams. The results of ultimate and proximate analyses as well as the heat of combustion and calorific values of coal samples prepared according to the standard PN-G-04506:1996 are given in Table 2. The samples were analyzed in the accredited Laboratory of Solid Fuels Quality Assessment of the Central Mining Institute, in accordance with the relevant standards in force: PN-G-04511:1980 (total moisture), PN-G-04560:1998 and PN-ISO 1171:2002 (ash), PN-G-04516:1998 and PN ISO-562:2000 (volatiles), PN-G-04513:1981 (heat of combustion, calorific value), PN-G-04584:2001 and PN-ISO 334:1997 (total and pyritic sulfur), and PN-G-04571:1998 (carbon content).

The determination of trace elements was performed in the Department of Environment Engineering of the Central Mining Institute. Coal samples were pretreated before analyses according to the standard ISO 1171:2002. They were combusted in a muffle furnace at 815°C. Next 3 g of produced ashes were palletized with graphite. The content of Hg in samples was determined with a high-temperature combustion technique coupled with cold vapor generation atomic absorption spectrometry (CVAAS) and gold amalgamation method—2000 Nippon Instrument Corporation. The contents of the remaining trace elements were determined with wavelength dispersive X-Ray fluorescence spectrometer Rigaku ZSX Primus.

## 4. Results and Discussion 

### 4.1. Trace Elements Content

The concentrations of trace elements in coal samples of selected zones of the USCB are presented in Table 3. Coal samples of the eastern and the western zones of the USCB are characterized by relatively higher average contents of Hg, As, Cd, Cr, Pb, Rb, V, and Zn than the remaining coal samples. The highest average concentrations of As, Cd, Pb, and Zn are observed for samples of the western part (15.94, 0.90, 131.57, and 147.52 ppm, resp.), whereas the highest average contents of Cr, Rb, and V are reported for coal samples of the eastern zone of the USCB (26.88, 32.86, and 70.10 ppm, resp.).

The geochemical characteristic of the USCB coals indicates that the highest concentrations of Cr are observed for samples of mudstone series and Upper Silesian sandstone series. The average content of Cr in Upper Silesian coals is higher than the values reported worldwide (9–13 ppm) [30, 31]. The variations in concentrations of this element in coal seams of particular lithostratigraphic series were insignificant (Table 3).

The average content of Zn in tested coal samples of USCB (111.19 ppm) is higher than the values reported worldwide (18–26 ppm) [30, 31]. Concentrations of Zn in tested coal samples vary across the USCB (from 78.52 ppm in central zone to 147.52 ppm in western zone of the USCB). The highest contents of Zn, locally exceeding 150 ppm, are reported for shallowly buried coal seams. The concentrations of Zn decrease with increasing depth of coal seams in the eastern zone of the USCB, which may be attributed to the postgenetic enrichment of coal in Zn, resulting from the occurrence of Zn-Pb ores in the Triassic overburden. The Zn-enrichment of coal seams of the western zone of the USCB may result from the raised geothermal gradient, typical for this region, and magmatism accompanied by various forms of mineralization (Table 3).

The average content of Pb in coals of the western zone of the USBC (131.57 ppm) is significantly higher than the values reported for the remaining zones (16.58–27.59 ppm) and worldwide (22–28 ppm) [30, 31]. The highest contents of Pb are observed for coals sampled from shallowly buried coal seams. The content of Pb decreases with the depth of coal seams, for example, in coal seams of sandstone series the concentration of Pb is approximately 77 ppm, in mudstone series 57 ppm, and in paralic series 39 ppm. The Pb-enrichment of coals of the eastern zone of the basin is caused by Zn and Pb ores deposited in Triassic formations. The highest contents of Pb are reported in the western zone of the basin. High concentrations of Pb are reported for deeper paralic series and low for Upper Silesian sandstone series, which may be attributed to the postgenetic mineralization related to the magmatism.

The average content of V in tested coal samples (52.34 ppm) is higher than the values reported for world coals (25–35 ppm) [30, 31]. The variations in concentrations of V for coals of various lithostratigraphic series are insignificant. The lowest values are observed for coal seams of Cracow sandstone series and high values for samples of the western zone of the basin.

Coal samples of the western zone of the USCB are also characterized by the highest average concentrations of Ba and Ni (386.30 and 28.34 ppm), whereas coal samples of the eastern zone of the USCB are unique due to the highest average content of Mn (157.69 ppm). The highest average Hg content is observed for coal samples of the eastern and the western zones of the USCB (0.03 ppm) (Table 3).

The lowest average contents of Ba, Co, Cr, Cu, Ni, Rb, Sr, and V (142.66, 5.44, 10.73, 17.47, 12.14, 8.04, 121.51, and 25.24 ppm) are observed for coal samples of the northern zone, whereas the lowest contents of Hg and Mn (0.01 and 69.37 ppm, resp.) are reported for coal samples of the southern zone of the USCB (Table 3).

Concentration of Ba in coal samples shows significant lateral variations resulting from the geological conditions. The average content of Ba in coal samples (257.10 ppm; Table 3) is higher than the values reported for world coals (111–149 ppm) [30, 31]. The highest concentrations of Ba are reported for coal seams of the western zone of the basin (386.30 ppm), in particular for coal seams located close to large and deep sinks filled with Miocene formations of saline sediments and evaporites. Barite veins are observed in coal seams of coal mines located in this area of the basin as well as deposition of barite from mine water.

The average concentration of Ni in coal samples is 21.21 ppm, which is slightly higher than the values reported worldwide (14–18 ppm) [30, 31]. The highest contents of Ni are observed for coals of mudstone series and Upper Silesian sandstone series and the lowest for samples of Cracow sandstone series (Table 3).

The average content of Mn in tested coal samples of the USCB amounts to 116.97 ppm (Table 3) and is higher than the values reported worldwide (79–111 ppm) [30, 31]. The lowest average concentration of this element is reported for coal seams of Cracow sandstone series, medium for coals of mudstone series and Upper Silesian sandstone series, and the highest for coals of paralic series. Significant enrichments of coals in Fe (60–70% Fe_2_O_3_ in ash) are observed locally within the paralic series, often accompanied by high concentration of Mn (this concerns mainly nonpyritic iron).

Coal samples provided by coal mines located in southern and western zones of the USCB are characterized by high average concentration of Sb (1.28 and 1.23 ppm, resp.). Furthermore, the highest average contents of Co, Cu, and Sr are observed for samples of the southern zone of the USCB (11.86, 32.20 and 257.42 ppm, resp.) (Table 3).

The uniqueness of coal samples collected in the central zone of the USCB was attributed to the lowest average concentrations of As, Cd, Pb, Sb, and Zn (2.03, 0.41, 16.58, 0.85, and 78.52 ppm, resp.) among the analyzed samples.

The average content of Co in tested coal samples is 8.57 ppm, which is higher than the values reported worldwide (4.9–5.5 ppm) [30, 31]. The variations in content of Co between coals of various lithostratigraphic series are not significant. High concentrations of Co are observed for coals of the southern and western zones of the USCB, and the lowest concentrations are observed for samples of the northern and central zones coal seams (Table 3).

The average content of Cu in Upper Silesian coals (25.69 ppm) is similar to the values reported for world coals (16–20 ppm) [30, 31]. The highest content of this element is reported for coal seams of Upper Silesian sandstone series and the lowest for Cracow sandstone series (Table 3). Slight Cu-enrichment is observed for coal seams of the south-western zone of the USCB, similar to the case of Zn and Pb. The average Cu content in coals of the southern zone increases with increasing depth of coal seams and amounts to 23 ppm for mudstone series, 41 ppm for Upper Silesian sandstone series, and 92 ppm for paralic series. The mechanism of the Cu-enrichment is probably similar to the one proposed for Zn- and Pb-enrichment.

### 4.2. Hierarchical Clustering Analysis

Coal samples provided by coal mines located in particular zones of the USCB differed both in terms of basic physical and chemical parameters and the contents of trace elements. The hierarchical clustering analysis (HCA) was applied to investigate the similarities and dissimilarities between the tested samples [20–26, 32]. The analyzed data set was organized in a matrix **X**(44 × 23). The rows of the matrix represent the tested coal samples provided by coal mines located in various zones of the USCB, whereas columns represent 23 parameters, including basic physical and chemical parameters and contents of trace elements (see Table 4). The data set was standardized since the measured parameters differed significantly in their ranges:

(1) $$\begin{array}{c} x_{ij}=\frac{x_{ij}-{\overset{-}{x}}_{j}}{s_{j}}, \end{array}$$

where ${\overset{-}{x}}_{j}$, *s*_*j*_ denote the mean of the *j*th column and its standard deviation, respectively.

**Figure 4:**
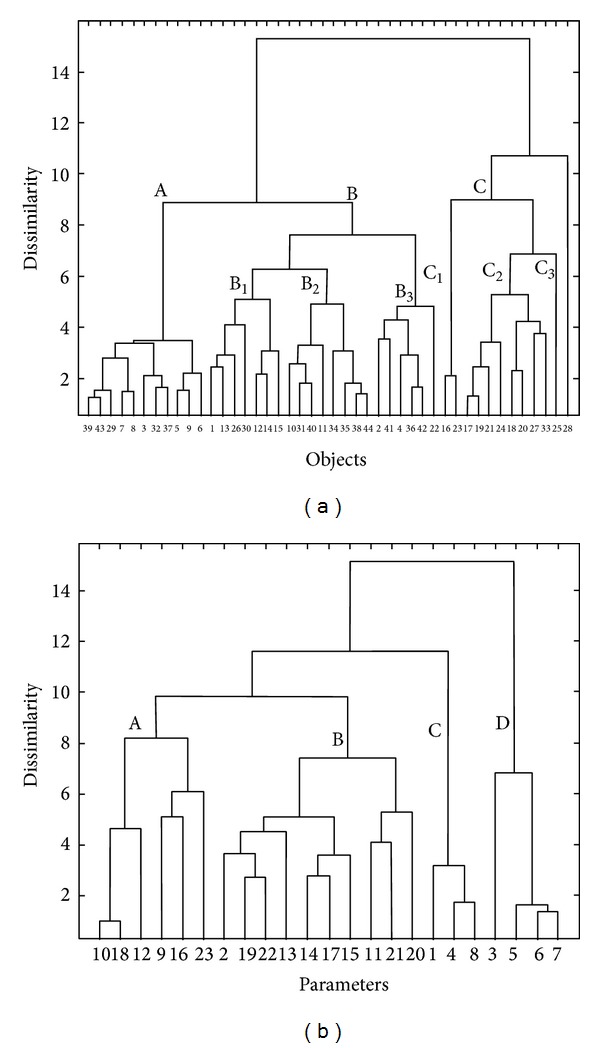
Dendrograms of (a) hard coal samples (objects) in the space of the 23 measured parameters (listed in Table 4) and (b) parameters in the space of 44 objects based on the Ward linkage method using Euclidean distance as the similarity measure.

The HCA helped reveal the internal data structure and thereof its clustering tendency. It enabled the analysis of the data structure by tracing the similarities/dissimilarities between objects (hard coal samples) in the parameters space and parameters in the objects space. The results were presented in a form of dendrograms, of which* OX* axis showed the sequence of objects/parameters clustering and* OY* axis determined the similarity/dissimilarity between them. The HCA methods differ in terms of parameters similarity measure applied and ways of cluster linking. The results presented in the paper were based on the Euclidean distance and the Ward linkage algorithm (see Figure 4). The Ward linkage method was based on the inner squared distance of clusters, so that at each stage these two clusters were merged, for which the minimum increase in the total within-group error sums of squares was observed. The dendrogram revealed the data structure (i.e., the subgroups of hard coal samples), but it allowed no interpretation of the observed patterns in terms of the parameters. To solve this problem a color map of the experimental standardized data sorted according to the order of objects and parameters observed on the dendrograms was used [32]. Simultaneous interpretation of the dendrogram of objects with the color map of the experimental data allowed more in-depth analysis of the relationships between hard coal samples from various zones of the USCB and measured parameters.

The dendrogram presented in Figure 4(a) enabled to distinguish three clusters: A, B, and C and one nonclustered object no. 28. Cluster A grouped six coal samples provided by coal mines located in the northern zone of the USCB (objects nos. 3, 5–9), one sample of the western zone (objet no. 29) and four samples of the central zone of the USCB (objects nos. 32, 37, 39, and 43, resp.). In cluster B, all samples of the southern zone (objects nos. 10–15), the remaining samples of the northern zone (objects nos. 1, 2, and 4), one sample of the eastern zone (object no. 22), two samples of the western zone (objects nos. 30 and 31), and the remaining samples of the central zone (except for sample no. 33) (objects nos, 34–36, 38, 40–42, and 44) were collected. Cluster C was composed of samples of the eastern zone (objects nos. 16–21, 23, and 24), two samples of the western zone (objects nos. 25 and 27), and one sample of the central zone of the USCB (object no. 33). In the main clusters B and C, additional substructures were distinguished. Namely, in cluster B, the following three subclusters of objects were observed:

- subcluster B1: collecting one sample of the northern zone (object no. 1), four samples of the southern (objects nos. 12–15), and two samples of the western zones of the USCB (objects nos. 26 and 30),
- subcluster B2: composed of two samples of the southern zone (objects nos. 10 and 11), one sample of the western zone (object no. 31), and five samples of the central zone of the USCB (objects nos. 34, 35, 38, 40, and 44), and
- subcluster B3: including two samples of the northern zone (objects nos. 2 and 4), one sample of the eastern zone (object no. 22), and three samples of the central zone of the USCB (objects nos. 36, 41, and 42, resp.).

In cluster C three subclusters (C1, C2, and C3) were distinguished. Subcluster C1 included two samples of the eastern zone of the USCB (objects nos. 16 and 23),,subcluster C2 included six samples of the eastern zone (objects nos. 17–21 and 24) and one sample of the western and central zones (objects nos. 27 and 33, resp.), and subcluster C3 was composed of one sample of the western zone of the USCB (object no. 25).

The dendrogram constructed for the basic physical and chemical parameters and trace elements contents (see Figure 4(b)) revealed four main groups:

- group A: including parameters nos. 9, 10, 12, 16, 18, and 23, which represent the contents of Hg, As, Cd, Mn, Pb, and Zn, respectively,
- group B collecting parameters nos. 2, 11, 13–15, 17, and 19–22, which represent the contents of ash, Ba, Co, Cr, Cu, Ni, Rb, Sb, Sr, and V, respectively,
- group C, composed of parameters nos. 1, 4, and 8, which represent contents of total moisture, total sulfur, and pyritic sulfur, respectively, and
- group D, including parameters nos. 3 and 5–7, which represent contents of volatiles, heat of combustion, calorific value, and carbon content, respectively.

The dendrogram of hard coal samples of various zones of the USCB presented the data structure but did not allow investigation of the observed patterns in terms of the original parameters. This disadvantage was overcome with the use of the color map of the standardized experimental data, sorted according to the specific order of objects (hard coal samples) and parameters adopted from the above described dendrograms [32].

**Figure 5:**
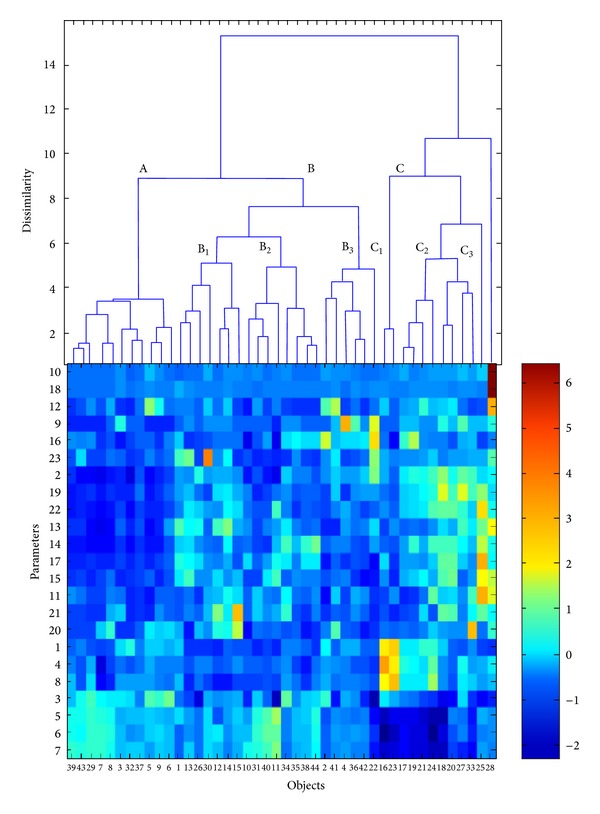
Dendrogram of 44 hard coal samples from various coal mines of the USCB (objects) in the space of the 23 measured parameters with the color map of the studied data sorted according to the Ward linkage method.

The analysis of the dendrogram of 44 objects in the space of the 23 parameters sorted according to the Ward linkage method with the data color map allowed more in-depth investigation of the resulting clustering tree (see Figure 5).

Coal samples collected in cluster A were characterized by the lowest contents of Co, Cr, Rb, and V (parameters nos. 13, 14, 19, and 22), low contents of ash, Cu, and Ni (parameters nos. 2, 15, and 17), and higher content of volatiles (parameter no. 3) than the remaining coal samples. Furthermore, the uniqueness of samples of northern, western, and central zones of the USCB (objects nos. 7, 8, 29, 39, and 43) was observed, resulting from high heat of combustion, calorific values, and carbon content (parameters nos. 5–7). Furthermore, one sample of the northern zone of the USCB (object no. 5) was characterized by the highest content of Cd (parameter no. 12).

All coal samples grouped in cluster B differed from the remaining tested samples mainly due to relatively low contents of total moisture, pyritic sulfur, and total sulfur (parameters nos. 1, 8, and 4).

Subcluster B1 was characterized by relatively high Co content (parameter no. 13), subcluster B2 by the lowest Zn content (parameter no. 23) and high heat of combustion, calorific value, and content of carbon (parameters nos. 5, 6, and 7), whereas subcluster B3 was unique due to high contents of Hg and Mn (parameters nos. 9 and 16). Coal samples of the southern and western zones of the USCB (objects nos. 15 and 30) were unique because of the highest contents of Sr and Zn (parameters nos. 21 and 23, respectively). Furthermore, coal samples of the northern and eastern zones (objects nos. 4 and 22) differed from the remaining samples due to the highest content of Hg (parameter no. 9). Moreover, the coal sample of the eastern zone of the USCB (object no. 22) was characterized by high contents of ash, Mn, and Zn (parameters nos. 2, 16, and 23, resp.).

The samples of cluster C, collecting samples of the eastern (objects nos. 16–21, 23, and 24), western (objects nos. 25 and 27), and central (object no. 33) zones of the USCB differed from the remaining samples due to the highest contents of ash, Cr, Rb, and V (parameters nos. 2, 14, 19, and 22) and relatively low values of volatiles, heat of combustion, calorific value, and carbon content (parameters nos. 3 and 5–7, resp.). Furthermore, the samples of the eastern zone (objects nos. 16 and 23) collected in subcluster C1 were characterized by the highest contents of total moisture, total sulfur, and pyritic sulfur (parameters nos. 1, 4, and 8). Coal samples of the eastern, western, and central zones (objects nos. 17–21, 24, 27, and 33, resp.), grouped in subcluster C2 were characterized by high contents of ash and Rb (parameters nos. 2 and 19), whereas the uniqueness of one sample of the western zone (object no. 25—see subcluster C3) resulted from the highest contents of Ba, Cr, Cu, Ni, and V (parameters nos. 11, 14, 15, 17, and 22) among all the tested samples.

The uniqueness of the nonclustered sample of the western zone of the USCB (object no. 28) was caused by the extremely high contents of As, Cd, and Pb (parameters nos. 10, 12, and 18) and low contents of total moisture and volatiles (parameters nos. 1 and 3) in comparison with the remaining samples.

## 5. Conclusions

The detailed analysis of the average trace elements concentrations in the tested coal samples of the northern, southern, western, eastern, and central zones of the USCB allowed us to draw the following conclusions.

(1) Coal samples of the eastern and western zones of the USCB were characterized by high average contents of Hg, As, Cd, Cr, Pb, Rb, V, and Zn. The highest average concentrations of As, Cd, Pb, and Zn were observed for samples of the western zone of the USCB.

The average content of Zn in tested coal samples of the USCB was higher than the values reported worldwide. Zn content varied across the USCB and decreased with increasing depth of coal seams in the eastern zone of the USCB, which may be attributed to the postgenetic enrichment of coal in Zn, resulting from the occurrence of Zn-Pb ores in the Triassic overburden. The Zn-enriched coal seams of the western zone of the USCB may result from the raised geothermal gradient, magmatism accompanied, and various forms of mineralization.

The average content of Pb in coals of the western zone of the USBC was significantly higher than the values reported for the remaining zones and worldwide. The content of Pb decreased with the depth of coal seams. The Pb-enrichment of coals of the eastern zone of the basin may result from the deposition of Zn and Pb ores in Triassic formations. High concentrations of Pb were reported for deeper paralic series and low concentrations were reported for Upper Silesian sandstone series, which may be attributed to the postgenetic mineralization related to the magmatism.

(2) The highest average concentrations of Cr, Rb, and V were reported for coal samples of the eastern zone. The average content of Cd in tested samples was lower and of Cr was higher than the values reported worldwide. The variations in the concentration of these elements in coal seams of particular lithostratigraphic series were insignificant. The average content of V in tested coal samples was higher than the values reported for world coals. The variations in concentrations of V for coals of various lithostratigraphic series were insignificant. The lowest values were observed for coal seams of Cracow sandstone series and high values for samples of the western zone of the basin.

(3) The highest average concentrations of Ba and Ni were observed for coals of the western zone. Concentration of Ba in coal samples tested showed significant lateral variations resulting from the geological conditions. The average content of Ba in tested coal samples was higher than the values reported for world coals. The highest concentrations of Ba were reported for the area, where barite veins are observed in coal seams as well as deposition of barite from mine water. The average concentration of Ni in tested coal samples was slightly higher than the values reported worldwide.

(4) Coal samples of the northern zone of the USCB were characterized by the lowest average Ba, Co, Cr, Cu, Ni, Rb, Sr, and V contents. The average content of Co in tested coal samples was higher than the values reported worldwide. The differences in content of Co between coals seams of various lithostratigraphic series were not significant. The average content of Cu in Upper Silesian coals was similar to the values reported for world coals. Slight Cu-enrichment was observed for coal seams of the south-western zone of the USCB. The average Cu content in coals of the southern zone increased with increasing depth of coal seams and the mechanism of the Cu-enrichment was probably similar to the one proposed for Zn and Pb.

(5) Coal samples of the southern zone of the USCB were characterized by the lowest contents of Hg and Mn. The average content of Mn in tested coal samples was higher than the values reported worldwide. Significant enrichments of coals in Fe were observed locally within the paralic series, often accompanied by high concentration of Mn.

(6) The lowest average concentrations of As, Cd, Pb, Sb, and Zn were observed for coal samples collected in the central zone of the USCB.

## Figures and Tables

**Table 1:** Characteristics of hard coal resources in Poland in 2012 [2].

	No. of deposits	Geological resources [mln Mg]		Industrial
		Balanced	Off balance	
Total	146	48,225	18,302	4,210

of which resources of exploited deposits:				
Operating plants	51	19,130	6,507	1,178

of which resources of off balance deposits:				
Total	**52**	**25,139**	**11,173**	—
Deposits explored	34	11,423	3,619	—
Deposits preexplored	18	13,716	7,553	—

of which resources of abandoned deposits:				
Total	43	3,955	621	—

**Table 2:** Values of basic physical and chemical parameters of hard coals of the Upper Silesian Coal Basin (USCB).

Zone of the USCB	Coal mine no.	Total moisture, *W* ^*a*^ [%]	Ash, *A* ^*a*^ [%]	Volatiles, *V* ^*a*^ [%]	Heat of combustion, *Q*_*s*_^*a*^ [kJ/kg]	Calorific value, *Q*_*i*_^*a*^ [kJ/kg]	Carbon, C_*t*_^a^ [%]	Total sulfur, S_*t*_^a^ [%]	Pyritic sulfur, S_p_^a^ [%]
Northern	1	5.01	15.36	28.53	28,681	23,059	64.37	0.73	0.51
	2	5.81	14.14	32.13	25,663	22,989	64.12	0.82	0.44
	3	5.36	9.40	31.03	27,650	25,522	69.24	0.67	0.43
	4	4.30	15.27	29.68	26,605	24,350	67.41	0.71	0.36
	5	5.13	7.52	32.72	28,522	25,720	68.32	0.88	0.41
	6	4.32	10.24	33.54	27,884	25,627	69.39	0.75	0.29
	7	2.24	7.13	31.30	30,339	28,247	75.89	0.24	0.03
	8	2.44	8.22	31.14	29,954	27,738	74.23	0.53	0.13
	9	4.86	8.90	32.26	28,056	26,317	70.85	0.73	0.33

Southern	10	2.35	8.07	32.05	30,101	27,954	74.53	0.62	0.08
	11	1.42	6.90	25.22	33,107	29,569	80.75	0.66	0.09
	12	1.40	15.89	29.07	25,508	26,688	69.89	0.58	0.08
	13	1.74	14.42	27.76	27,350	24,339	70.62	0.59	0.08
	14	2.75	15.79	29.55	27,300	24,348	66.19	0.63	0.10
	15	2.38	14.48	29.91	28,598	25,550	68.91	0.77	0.19

Eastern	16	11.41	15.51	31.15	22,119	17,658	55.29	2.15	1.25
	17	5.74	18.86	28.86	23,425	20,734	58.19	1.20	0.60
	18	4.85	26.38	26.26	21,834	19,854	54.43	0.76	0.39
	19	5.66	21.02	28.59	23,387	19,990	56.65	1.09	0.60
	20	2.80	20.25	26.51	26,503	22,889	62.16	0.64	0.24
	21	7.03	20.71	27.83	22,531	19,925	56.31	1.00	0.62
	22	2.78	25.59	25.04	23,853	21,764	59.12	0.50	0.22
	23	11.97	11.72	29.76	23,298	19,109	56.69	1.92	1.41
	24	6.94	22.43	27.54	22,009	17,929	54.91	1.40	1.02

Western	25	1.95	18.19	26.47	27,636	23,807	67.80	0.88	0.41
	26	1.79	21.21	25.91	26,330	23,508	64.66	0.74	0.32
	27	2.56	23.95	30.84	26,306	23,872	60.66	1.15	0.60
	28	1.74	19.46	27.03	27,116	24,471	65.84	1.05	0.49
	29	2.44	7.84	32.63	30,616	28,510	76.25	0.89	0.56
	30	1.64	15.25	29.88	28,772	26,259	71.55	0.71	0.18
	31	1.39	11.54	30.47	30,399	28,505	75.40	0.62	0.18

Central	32	6.72	6.20	30.83	28,094	24,246	70.91	0.61	0.40
	33	3.68	22.54	27.43	23,912	21,503	58.86	0.83	0.53
	34	3.50	17.02	33.28	26,744	23,915	64.75	0.78	0.43
	35	3.32	13.83	29.35	27,932	25,439	69.00	0.56	0.32
	36	4.27	15.35	29.07	26,384	24,736	65.44	0.60	0.33
	37	3.49	11.13	29.05	28,218	24,855	70.87	0.68	0.40
	38	2.67	13.30	30.27	28,302	25,492	69.34	0.39	0.16
	39	2.66	9.77	30.26	30,101	27,420	76.05	0.72	0.48
	40	2.15	8.53	28.33	31,484	29,395	77.24	0.57	0.23
	41	2.85	17.56	26.93	26,992	24,534	66.82	0.79	0.28
	42	2.82	17.05	27.15	26,820	24,474	66.18	0.57	0.29
	43	3.40	7.66	31.62	29,988	27,192	74.93	0.57	0.24
	44	1.90	11.04	30.64	30,229	27,635	73.60	0.67	0.35

**Table 3:** Concentrations of trace elements in hard coal samples of the Upper Silesian Coal Basin (USCB).

Zone of the USCB	Coal mine	Hg [ppm]	As [ppm]	Ba [ppm]	Cd [ppm]	Co [ppm]	Cr [ppm]	Cu [ppm]	Mn [ppm]	Ni [ppm]	Pb [ppm]	Rb [ppm]	Sb [ppm]	Sr [ppm]	V [ppm]	Zn [ppm]
Northern	1	0.01	0.46	149.71	0.61	15.59	26.72	35.62	154.09	36.01	62.42	21.57	1.23	97.66	44.61	222.19
	2	0.01	5.49	47.45	1.58	2.95	14.64	14.87	314.18	5.78	16.60	7.80	0.88	81.92	25.23	101.90
	3	0.05	4.63	188.13	0.19	5.83	8.22	9.78	38.91	11.04	26.06	4.70	0.38	204.14	24.46	75.33
	4	0.11	3.82	138.65	0.31	8.18	16.98	21.01	165.09	9.74	6.26	7.33	0.69	108.82	56.18	53.25
	5	0.00	9.13	57.27	1.88	2.02	5.13	16.61	96.27	7.69	40.11	2.66	1.58	65.40	15.41	87.56
	6	0.00	1.77	183.71	0.48	8.63	11.48	15.52	107.30	13.94	20.52	13.24	1.54	119.03	26.15	79.06
	7	0.00	0.63	120.84	0.39	1.18	3.72	14.18	65.85	7.69	1.34	2.60	1.40	71.55	7.59	51.92
	8	0.01	1.22	218.81	0.82	1.55	3.82	19.61	77.60	7.13	7.85	6.19	1.97	188.78	12.77	78.41
	9	0.00	3.79	179.33	1.22	3.02	5.85	10.07	59.71	10.29	19.94	6.32	1.41	156.26	14.72	55.59

Southern	10	0.01	2.93	177.94	0.08	7.53	15.36	13.90	25.06	28.96	12.29	16.51	0.08	114.88	39.13	54.99
	11	0.00	2.28	191.27	0.55	8.63	23.67	45.40	23.53	33.05	18.63	14.56	0.76	179.75	95.57	22.01
	12	0.02	1.59	302.72	0.53	15.20	13.93	33.69	101.29	17.16	18.65	30.09	1.91	360.51	47.84	101.56
	13	0.02	0.58	205.41	0.58	12.69	27.39	46.99	65.73	32.00	25.66	26.09	0.14	145.74	72.36	253.70
	14	0.02	5.39	419.58	0.97	17.76	32.29	23.95	98.68	22.74	28.74	33.05	1.84	232.33	76.26	183.88
	15	0.01	4.78	355.85	0.29	9.34	14.41	29.25	101.94	11.95	18.24	23.39	2.97	511.29	71.10	42.79

Eastern	16	0.01	2.53	128.59	0.95	4.74	13.36	24.41	105.90	29.02	29.31	15.07	0.74	106.06	46.28	126.24
	17	0.03	7.19	86.99	0.73	5.82	21.27	28.31	214.78	21.48	22.75	28.47	0.75	84.33	66.08	106.83
	18	0.05	5.98	341.49	0.92	11.34	37.63	48.97	108.63	43.21	35.92	66.16	1.06	327.29	104.19	97.07
	19	0.03	3.09	108.42	0.78	6.18	36.59	32.29	290.80	20.83	40.10	31.11	0.63	100.68	69.59	101.31
	20	0.03	6.07	356.88	1.10	9.31	38.38	53.22	84.11	42.02	19.46	44.86	1.01	317.16	104.06	118.01
	21	0.02	3.85	206.49	1.41	5.90	23.30	38.21	120.93	19.82	27.63	36.24	0.90	223.55	74.17	113.31
	22	0.09	0.77	96.99	0.51	11.26	20.22	6.40	356.47	4.35	26.87	22.78	0.38	67.05	34.29	278.16
	23	0.01	3.85	242.03	0.46	7.78	12.38	8.43	29.48	10.01	15.11	11.59	0.66	65.23	33.42	92.02
	24	0.02	5.61	545.76	0.90	7.79	38.81	27.24	108.09	27.40	31.18	39.48	1.35	109.12	98.80	142.48

Western	25	0.03	10.37	881.83	0.36	16.92	53.12	71.50	71.68	69.13	24.74	55.13	0.73	332.03	156.46	122.99
	26	0.01	0.64	112.63	0.42	13.36	19.51	22.91	75.30	28.42	18.45	15.91	1.27	115.38	65.96	44.75
	27	0.06	9.22	238.18	0.48	15.09	31.49	17.72	135.20	26.70	28.86	66.58	0.96	143.82	90.29	110.17
	28	0.05	79.25	720.91	2.72	23.02	32.69	61.81	110.70	31.78	822.66	31.26	2.01	245.66	81.84	230.29
	29	0.00	1.04	136.48	0.63	1.57	4.20	9.27	117.11	8.43	6.88	6.04	0.43	73.46	9.21	52.84
	30	0.02	7.27	290.02	0.92	5.95	15.10	23.19	77.28	20.03	11.69	8.08	0.99	191.84	50.54	439.25
	31	0.01	3.80	324.06	0.74	4.50	11.26	13.61	78.25	13.85	7.66	4.15	0.74	210.45	30.99	32.34

Central	32	0.01	0.49	83.73	0.14	3.04	2.85	10.18	75.97	7.91	4.92	3.10	0.38	65.14	5.34	40.93
	33	0.03	6.16	495.15	0.27	12.09	24.38	24.90	149.42	19.67	28.44	44.39	3.75	252.24	65.80	120.18
	34	0.02	4.75	405.11	0.29	11.80	38.20	16.49	171.82	29.61	22.51	19.82	0.54	284.08	43.57	42.45
	35	0.02	0.84	188.19	0.20	7.06	24.66	24.14	185.95	17.28	8.92	10.98	0.65	108.19	18.93	104.17
	36	0.06	2.54	289.43	0.52	6.38	12.66	11.80	170.81	13.18	28.62	20.56	0.57	156.73	39.30	64.55
	37	0.01	0.68	111.23	0.48	4.97	7.95	13.74	89.47	10.42	10.29	15.14	0.64	73.99	23.95	49.41
	38	0.01	1.00	170.01	0.21	5.52	36.13	21.78	164.93	31.06	18.81	14.19	0.17	79.27	32.87	48.50
	39	0.00	1.36	249.53	0.20	4.01	5.70	11.58	117.41	6.94	5.13	2.77	0.47	83.90	13.49	64.19
	40	0.01	0.97	209.99	0.29	7.02	8.83	35.46	131.12	21.06	14.99	9.04	0.67	154.29	30.65	34.78
	41	0.03	4.39	323.00	1.97	2.85	17.39	18.44	145.73	14.82	36.95	28.94	2.22	120.90	44.05	163.85
	42	0.02	0.79	240.17	0.24	7.13	10.11	4.99	175.04	11.52	17.52	21.79	0.20	118.02	26.90	86.77
	43	0.00	1.03	201.13	0.34	3.50	5.88	13.19	112.16	6.84	4.01	3.90	0.47	91.55	13.95	159.41
	44	0.01	1.33	234.48	0.18	6.50	43.72	22.43	167.24	30.79	14.37	12.82	0.38	143.56	31.21	41.62

**Table 4:** Parameters of hard coal samples of the Upper Silesian Coal Basin (USCB) analyzed with HCA.

No.	Parameter	Unit
1	Total moisture, *W* ^*a*^	[%]
2	Ash, *A* ^*a*^	[%]
3	Volatiles, *V* ^*a*^	[%]
4	Total sulfur, S_*t*_^a^	[%]
5	Heat of combustion, *Q*_*s*_^a^	[kJ/kg]
6	Calorific value, *Q*_*i*_^*a*^	[kJ/kg]
7	Carbon, C_*t*_^a^	[%]
8	Pyritic sulfur, S_p_^a^	[%]
9	Hg	[ppm]
10	As	[ppm]
11	Ba	[ppm]
12	Cd	[ppm]
13	Co	[ppm]
14	Cr	[ppm]
15	Cu	[ppm]
16	Mn	[ppm]
17	Ni	[ppm]
18	Pb	[ppm]
19	Rb	[ppm]
20	Sb	[ppm]
21	Sr	[ppm]
22	V	[ppm]
23	Zn	[ppm]
